# Evaluation of Amide Proton Transfer-Weighted Imaging for Risk Factors in Stage I Endometrial Cancer: A Comparison With Diffusion-Weighted Imaging and Diffusion Kurtosis Imaging

**DOI:** 10.3389/fonc.2022.876120

**Published:** 2022-04-14

**Authors:** Xingxing Jin, Ruifang Yan, Zhong Li, Gaiyun Zhang, Wenling Liu, Hongxia Wang, Meng Zhang, Jinxia Guo, Kaiyu Wang, Dongming Han

**Affiliations:** ^1^ Department of Magnetic Resonance Imaging (MRI), The First Affiliated Hospital, Xinxiang Medical University, Weihui, China; ^2^ Magnetic Resonance Imaging (MRI) Research China, General Electric (GE) Healthcare, Beijing, China

**Keywords:** endometrial cancer, amide proton transfer-weighted imaging, diffusion kurtosis imaging, diffusion-weighted imaging, risk factors

## Abstract

**Background:**

Endometrial cancer (EC) is one of the most common gynecologic malignancies in clinical practice. This study aimed to compare the value of diffusion-weighted imaging (DWI), diffusion kurtosis imaging (DKI), and amide proton transfer-weighted imaging (APTWI) in the assessment of risk stratification factors for stage I EC including histological subtype, grade, stage, and lymphovascular space invasion (LVSI).

**Methods:**

A total of 72 patients with stage I EC underwent pelvic MRI. The apparent diffusion coefficient (ADC), mean diffusivity (MD), mean kurtosis (MK), and magnetization transfer ratio asymmetry (MTRasym at 3.5 ppm) were calculated and compared in risk groups with the Mann–Whitney *U* test or independent samples *t*-test. Spearman’s rank correlation was applied to depict the correlation of each parameter with risk stratification. The diagnostic efficacy was evaluated with receiver operating characteristic (ROC) curve analysis and compared using the DeLong test. A multivariate logistic regression was conducted to explore the optimal model for risk prediction.

**Results:**

There were significantly greater MTRasym (3.5 ppm) and MK and significantly lower ADC and MD in the non-adenocarcinoma, stage IB, LVSI-positive, high-grade, and non-low-risk groups (all *p* < 0.05). The MK and MTRasym (3.5 ppm) were moderately positively correlated with risk stratification as assessed by the European Society for Medical Oncology (EMSO) clinical practice guidelines (*r* = 0.640 and 0.502, respectively), while ADC and MD were mildly negatively correlated with risk stratification (*r* = −0.358 and −0.438, respectively). MTRasym (3.5 ppm), MD, and MK were identified as independent risk predictors in stage I EC, and optimal predictive performance was obtained with their combinations (AUC = 0.906, sensitivity = 70.97%, specificity = 92.68%). The results of the validation model were consistent with the above results, and the calibration curve showed good accuracy and consistency.

**Conclusions:**

Although similar performance was obtained with each individual parameter of APTWI, DWI, and DKI for the noninvasive assessment of aggressive behavior in stage I EC, the combination of MD, MK, and MTRasym (3.5 ppm) provided improved predictive power for non-low-risk stage I EC and may serve as a superior imaging marker.

## Introduction

Endometrial cancer (EC) is one of the most common gynecologic malignancies in clinical practice, and 80% of newly diagnosed patients are in stage I (1, 2). According to the histological subtype, grade, International Federation of Gynecology and Obstetrics (FIGO) stage, and lymphovascular space invasion (LVSI), the European Society for Medical Oncology (ESMO) clinical practice guidelines classify stage I EC into low risk, intermediate risk, intermediate high risk, and high risk (3). For low-risk patients, lymphadenectomy is likely to lead to complications and increased care costs, thereby reducing their survival benefit, but in non-low-risk (intermediate-, intermediate-high-, and high-risk) patients, lymphadenectomy is necessary and effective (4). The histological subtype, grade, FIGO stage, and LVSI, which are obtained mainly by preoperative magnetic resonance imaging (MRI) and biopsy at present, are important factors for the risk stratification of stage I EC (3, 5). However, the accuracy of FIGO stage evaluation using conventional T1-weighted (T1W) and T2-weighted (T2W) MRI may be influenced by factors such as adenomyosis, leiomyomas, myometrial compression, and loss of the junctional zone (6, 7). In addition, biopsy has the disadvantages of invasiveness, inadequate sampling, and susceptibility to operator experience (8, 9). Therefore, it is of great interest to discover a noninvasive and effective means for assessing stage I EC risk factors for stratification, thus complementing existing methods.

Diffusion and molecular MR imaging techniques have been explored for the diagnosis and differentiation of EC. Diffusion-weighted (DW) MRI detects the diffusion movement of water molecules in tissues (10). Jiang et al. showed that diffusion-weighted imaging (DWI) can help differentiate EC from normal endometrial parenchyma (11). Diffusion kurtosis imaging (DKI), as an evolutionary technique of DWI, takes into account the non-Gaussian distribution of the diffusion movement of water molecules in the tissue and is considered as a more accurate imaging technique to characterize the microstructure of the lesion (12, 13). Amide proton transfer-weighted imaging (APTWI) is a molecular imaging method that utilizes the chemical exchange between amide protons and water molecules to quantify the mobile proteins and peptides in tissues (14). Yue et al. and Takayama et al. indicated that DKI and APTWI can play active roles in the histological grading assessment of EC (15, 16). Meng et al. found that both DKI and APTWI can be used in the diagnosis of EC with different clinical and histological types (17). Based on these results, we hypothesized that DWI-, DKI-, and APTWI-related parameters may be useful predictors of the risk stratification factors for stage I EC.

A few studies have briefly reported the role of these techniques in stage I EC risk stratification (18, 19). However, these studies either explored the application of only a single imaging technique or assessed only risk stratification without evaluating the risk stratification factors. The aims of this study were to compare the value of DWI, DKI, and APTWI in assessing the risk stratification factors for stage I EC, including histological subtype, grade, FIGO stage, and LVSI, and to explore the advantage of including multiple parameters from MRI in differentiating low-risk and non-low-risk stage I EC patients.

## Materials and Methods

### Study Population

**Figure 1:**
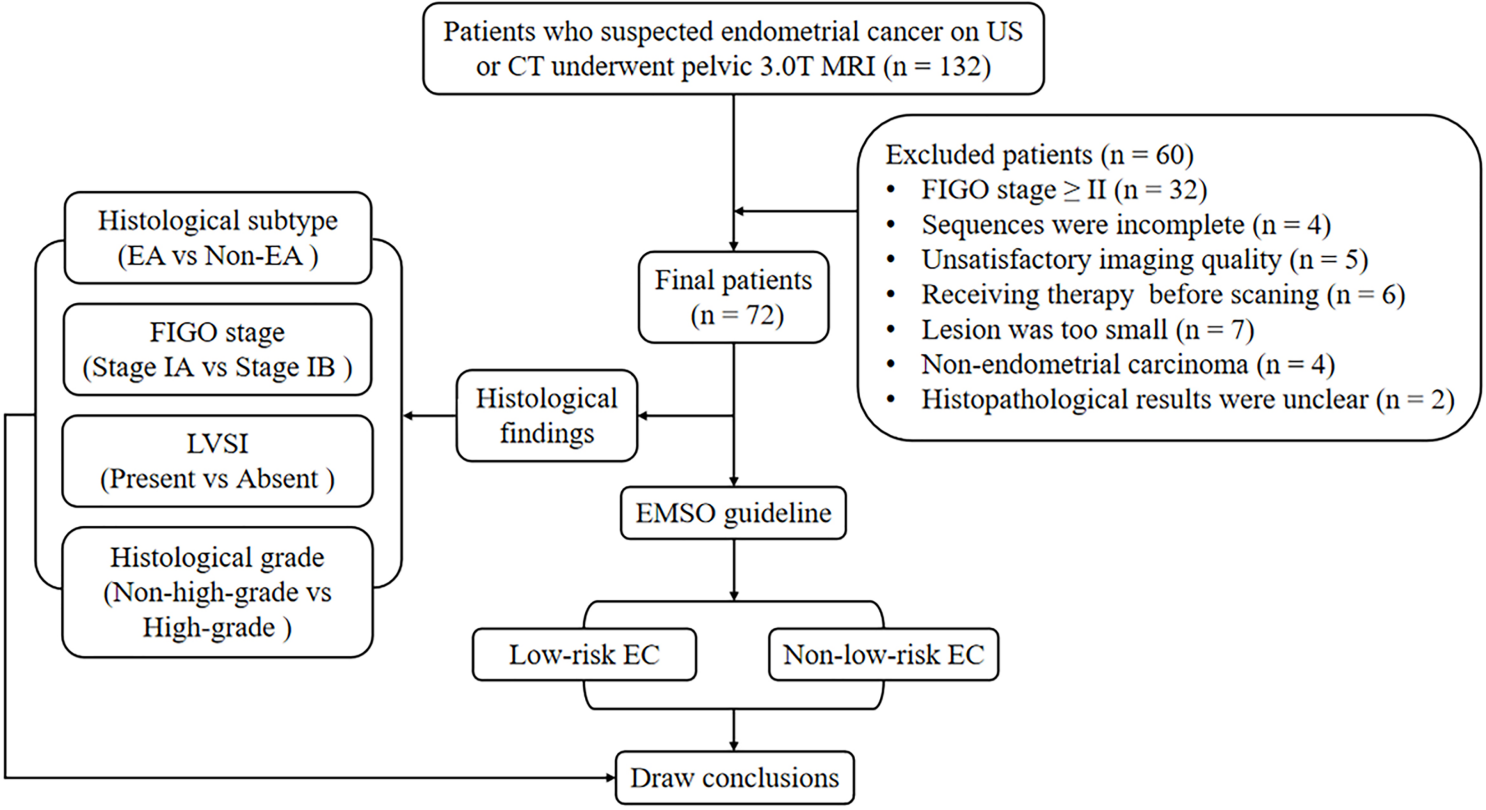
Flowchart of the present study.

The local institutional review board approved the present study, and all participants provided written informed consent. A series of 132 consecutive female patients with suspected EC on computed tomography (CT) or ultrasound (US) underwent pelvic MRI between July 2018 and June 2021. Sixty participants were excluded for the following reasons: 1) having FIGO stage II, III, or IV (*n* = 32); 2) having claustrophobia or other diseases or conditions that prevent them from completing all the sequences (*n* = 4); 3) inadequate imaging quality in DWI, DKI, or APTWI for analysis due to severe artifacts (*n* = 5); 4) received relevant treatment prior to scanning (*n* = 6); 5) having the largest area of the lesion <50 pixels (392 mm^2^) in the axial plane of DWI, DKI, or APTWI (*n* = 7); 6) histological findings of non-EC (*n* = 4); and 7) uncertain histological findings (*n* = 2). Ultimately, 72 patients were enrolled in the present study (**Figure 1**).

### MRI Protocols

All pelvic MRI examinations were acquired with a 3.0-T MRI system (Discovery MR750, GE Healthcare, Waukesha, WI, USA) using a 16-channel phased-array body coil. Participants were given 40 mg of hyoscine butylbromide (Buscopan; Boehringer, Ingelheim, Germany) intramuscularly or intravenously prior to the examination to minimize bowel motion. All participants were placed in the supine position with their feet first and a partially full bladder. Two-dimensional oblique axial (perpendicular to the long axis of the cervix) T1W imaging (T1WI), T2W imaging (T2WI), and DWI were performed first. Subsequently, all slices containing lesions were selected from the images from DWI, and their position, layer thickness, and layer spacing were copied to DKI and APTWI for the corresponding scans. Finally, a three-dimensional axial contrast-enhanced sequence was performed *via* intravenous injection (0.1 ml/kg, 2.0 ml/s) of gadopentetate dimeglumine (Gd-DTPA; Bayer Pharmaceutical, Berlin, Germany) using an automatic injector. Details of the protocol are provided in **Table 1**.

**Table 1:** Imaging protocol parameters.

Parameters	T1WI	T2WI	DWI	DKI	APTWI	Contrast-enhanced imaging
Sequence	2D FSE	2D FSE	2D SS-EPI	2D SS-EPI	2D EPI	3D LAVA
Orientation	Axial	Axial	Axial	Axial	Axial	Axial
Repetition time/echo time (ms)	605/8	5,455/109	6,000/60.5	2,500/58.9	3,000/12	4.2/2.1
Field of view (cm^2^)	36 × 36	36 × 36	36 × 36	36 × 36	36 × 36	36 × 36
Matrix	320 × 224	320 × 224	128 × 128	128 × 128	128 × 128	320 × 320
Bandwidth (Hz/pixel)	62.50	83.33	250	250	250	83.33
Slice thickness (mm)	5	5	5	5	5	1
No. of sections	20	20	20	Based on lesion size	Based on lesion size	80
No. of excitation	1	1	1 (*b* = 0)	2	1	0.7
			4 (*b* = 1,000)			
Diffusion encoding directions	–	–	1	30	–	–
Fat suppression	–	STIR	STIR	SPECIAL	STIR	FLEX
*b*-values (s/mm^2^)	–	–	0, 1000	0, 500, 1,000,1,500, 2,000	–	–
Respiratory compensation	Free	Free	Free	Free	Free	Breath holding
Scan time	1 min, 57 s	1 min, 33 s	1 min, 24 s	5 min, 28 s	2 min, 36 s (single slice)	9 s (each phase)

Saturation pulses (T_sat_) of 0.5 s and a saturation level of 2.0 μT were used to perform APTWI. A total of 52 frequencies, including a frequency 5000 Hz (3 times) away from the resonant frequency and 49 offsets ranging from -600 to +600 Hz with an interval of 25 Hz, were used for signal normalization of APTWI and z-spectrum scans. The water saturation shift reference (WASSR) was applied for B_0_ correction. The number of DKI diffusion gradient directions is 30.

T1WI, T1-weighted imaging; T2WI, T2-weighted imaging; DWI, diffusion-weighted imaging; DKI, diffusion kurtosis imaging; APTWI, amide proton transfer-weighted imaging; FSE, fast spin echo; SS-EPI, single-shot echo planar imaging; LAVA, liver acquisition with volume assessment; FLEX, flexible; STIR, short-inversion time (TI) recovery; SPECIAL, spectral inversion at lipids.

### Image Post-Processing

All images were transferred to the Advantage Workstation (version 4.6; GE Healthcare) and post-processed with the apparent diffusion coefficient (ADC), DKI, and amide proton transfer (APT) processing tools independently by two genitourinary radiologists (XJ and RY, with 7 and 15 years of experience, respectively.) who were unaware of each other’s outcomes and of the clinical and histological information. The DWI parameter was drawn from the following formula:


 (1) $$S_{b}/S_{0}=\text{exp}(-b\;\times \;\text{ADC})$$

where *b* is the diffusion sensitizing factor, *S*_0_ and *S*_b_ are the signal intensities (SIs) under zero and nonzero *b* values, respectively, and ADC is the apparent diffusion coefficient (10). The DKI parameter was derived from the following function:


(2) $$S_{b}=S_{0}\times \text{exp}(-b\;\times D_{\text{app}}+b^{2}\times D_{\text{app}}2\times K_{\text{app}}/6\;)$$

where *D*_app_ denotes the diffusion coefficient corrected for non-Gaussian bias and *K*_app_ denotes the degree of deviation from the Gaussian distribution. MD and MK reflect the average *D*_app_ and *K*_app_ values for all directions, respectively (12). The APTWI parameter was calculated using the following equation:


 (3) $$\mathrm{MTRasym}(3.5\;\text{ppm})=[S_{\text{sat}}(-3.5\;\text{ppm})-S_{\text{sat}}(+3.5\;\text{ppm})]/S_{0}$$

where *S*_sat_ and *S*_0_ denote the SIs obtained with and without selective saturation, respectively, and MTRasym (3.5 ppm) is the asymmetric magnetization transfer ratio at 3.5 ppm (14). With the DWI and contrast-enhanced images as references, the region of interest (ROI) of the lesion was manually delineated layer by layer along the inside of the tumor margin on axial T2WI, where areas with necrosis, apparent signs and hemorrhage artifacts, cystic degeneration, and blood vessels were avoided (17). All ROIs were automatically copied to each parameter map by the software to calculate the mean values.

### Histopathological Analysis

All lesion specimens were harvested through surgery with a median interval of 10 days (range, 1–24 days) between pelvic MRI examination and surgery. An experienced pathologist analyzed these specimens without knowledge of the clinical and imaging findings. The histological grade, subtype, and LVSI were confirmed by hematoxylin/eosin (HE) staining. The depth of myometrial invasion was assessed using the FIGO staging system (20). The new ESMO clinical practice guidelines were used to assess the risk stratification, and eventually, all included participants were classified into four groups: low, intermediate, high-intermediate, and high risk (3). Then, the low-risk stage I EC patients were categorized into the low-risk group and the intermediate, high-intermediate, and high-risk stage I EC patients, who are usually considered to have undergone lymphadenectomy, were categorized into the non-low-risk group (18).

### Statistical Analysis

The intraclass correlation coefficient (ICC) was calculated to assess inter-observer agreement (*r* < 0.40, poor; 0.40 ≤ *r* < 0.60, fair; 0.60 ≤ *r* < 0.75, good; and *r* ≥ 0.75, excellent) (21). After checking the normality of the data with the Shapiro–Wilk test, the Mann–Whitney *U* test was used for the comparison of non-normally distributed data (median and interquartile range) and the independent samples *t*-test used for the comparison of normally distributed data (mean ± standard deviation). The diagnostic efficacy of the different parameters was described by the area under the receiver operating characteristic (ROC) curve (AUC) and compared with DeLong analysis. A multivariate logistic regression was performed to explore the optimal differentiation performance with multiple parameters. The regression model was also verified using calibration curves with bootstrapping (1,000 samples) (22). Spearman’s rank correlation was applied to evaluate the correlation of each parameter with risk stratification (*r* ≥ 0.75, good; 0.50 ≤ *r* < 0.75, moderate; 0.25 ≤ *r* < 0.50, mild; and *r* < 0.25, little or none) (23). All analyses were performed by Stata (version 16.0; StataCorp, College Station, TX, USA) and MedCalc (version 15.0; MedCalc Software, Oostende, Belgium) software. A *p* < 0.05 was considered statistically significant.

## Results

### Basic Information

**Figure 2:**
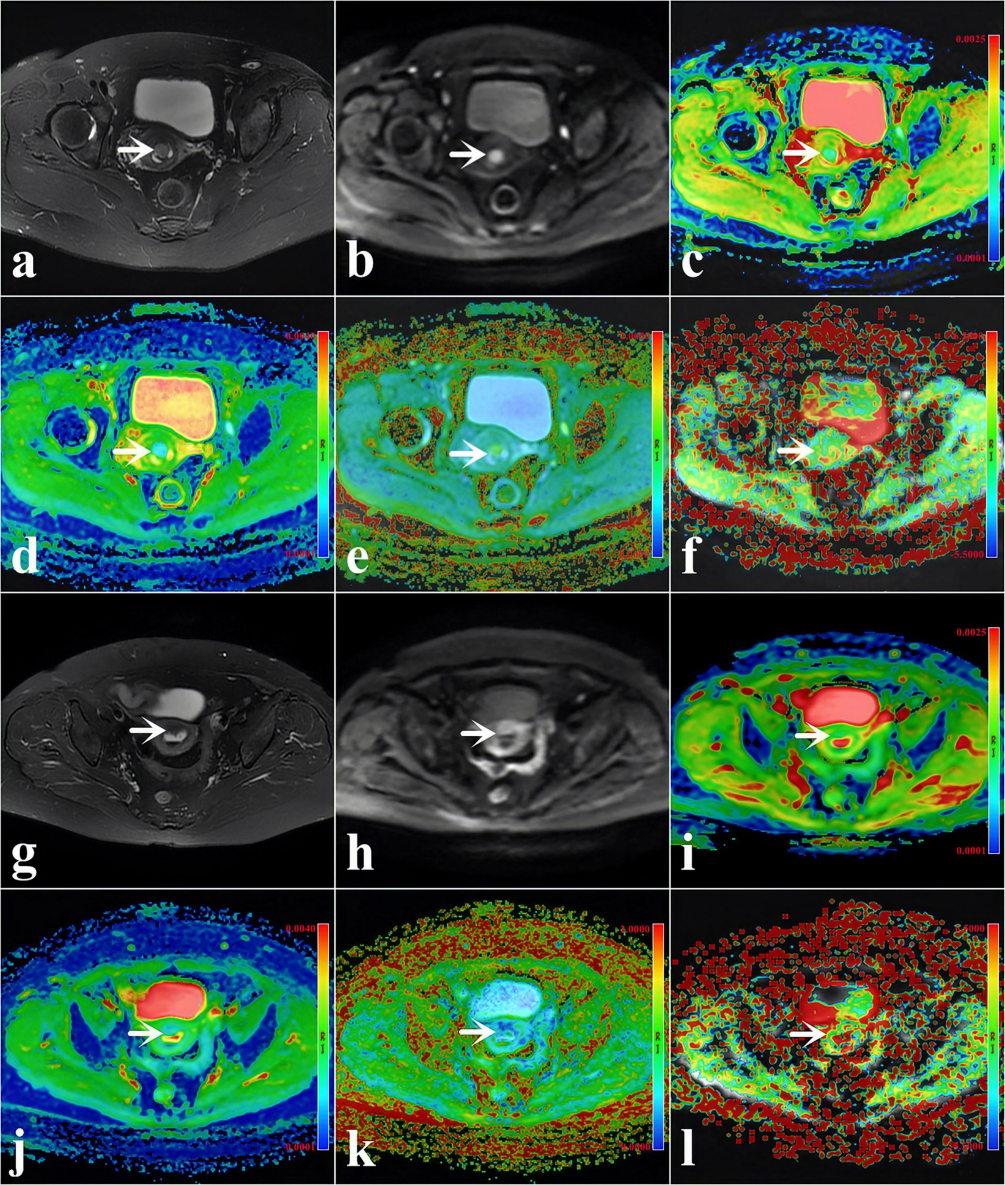
**(A–F)** A 48-year-old woman with low-risk endometrial cancer (EC) [*arrowheads*, endometrioid type; grade 1, stage IA, lymphovascular space invasion (LVSI)-negative]. **(G–L)** A 61-year-old woman with high-intermediate-risk EC (*arrowheads*, endometrioid type; grade 3, stage IA, LVSI-positive). **(A, G)** T2-weighted imaging (T2WI) maps (fat suppression). **(B, H)** Diffusion-weighted imaging (DWI) original maps (*b* = 1,000 s/mm^2^). **(C, I)** Pseudo-colored maps of the apparent diffusion coefficient (ADC). **(D, J)** Pseudo-colored maps of the mean diffusivity (MD). **(E, K)** Pseudo-colored maps of the mean kurtosis (MK). **(F, L)** Pseudo-colored maps of the magnetization transfer ratio asymmetry (MTRasym, at 3.5 ppm).

**Table 2** and **Figure 2** present the clinicopathological and imaging information of the patients, respectively.

**Table 2:** Clinicopathological features of the patients.

Variable	Data
Age (years), mean ± SD	58.89 ± 7.53
Maximum diameter (mm), mean ± SD	52.07 ± 15.31
FIGO stage, *n* (%)	
IA	44 (61.11)
IIB	28 (38.89)
Histologic subtype, *n* (%)	
Adenocarcinoma	67 (93.06)
Non-adenocarcinoma	5 (6.94)
Clear cell	3 (4.17)
Serous	2 (2.77)
Lymphovascular space invasion, *n* (%)	
Present	18 (25.00)
Absent	54 (75.00)
Histological grade, *n* (%)	
Grade 1	31 (43.06)
Grade 2	24 (33.33)
Grade 3	17 (23.61)
Risk stratification, *n* (%)	
Low	41 (56.94)
Intermediate	7 (9.72)
High-intermediate	12 (16.67)
High-risk group	12 (16.67)

### Consistency Between Two Radiologists for Quantification

The quantification parameters measured by the two radiologists showed excellent consistency, and the ICCs of ADC, MTRasym (3.5 ppm), MD, and MK were 0.896 (95% CI = 0.834–0.935), 0.844 (95% CI = 0.752–0.903), 0.881 (95% CI = 0.809–0.925), and 0.861 (95% CI = 0.778–0.913), respectively. The average results of the two radiologists were used for the final analysis.

### Assessment of Risk Factors

**Figure 3:**
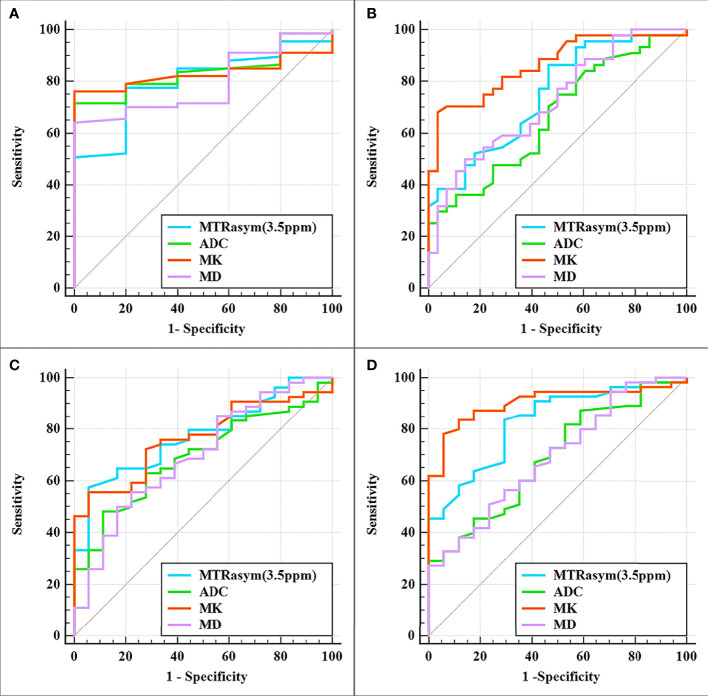
Curves showing each parameter using receiver operating characteristic (ROC) analysis for the differentiation of adenocarcinoma and non-adenocarcinoma **(A)**, stage IA and stage IB **(B)**, LVSI-positive and LVSI-negative **(C)**, and high-grade and non-high-grade **(D)** stage I endometrial cancer (EC). Details of the area under the curves and the 95% CIs of each index are shown in **Table 5**.

The differentiation of adenocarcinoma from non-adenocarcinoma in stage I EC showed significantly greater MTRasym (3.5 ppm) and MK and significantly lower ADC and MD in the non-adenocarcinoma group than those in the adenocarcinoma group (all *p* < 0.05). The AUCs of MD, MTRasym (3.5 ppm), MK, and ADC were 0.839, 0.793, 0.830, and 0.797, respectively. No statistically significant differences among these AUCs were found (**Tables 3**, **4** and **Figure 3A**).

**Table 3:** Comparison of the different parameters among different groups.

Parameters	MTRasym (3.5 ppm) (%)	ADC (×10^−3^ mm^2^/s)	MK	MD (×10^−3^ mm^2^/s)
Histological subtype				
Adenocarcinoma	3.54 ± 0.41	0.98 (0.92–1.04)	0.79 (0.76–0.83)	1.22 (1.10–1.27)
Non-adenocarcinoma	3.96 ± 0.30	0.90 (0.86–0.92)	0.85 (0.83–0.87)	1.13 (0.99–1.15)
*t*/*z* value	2.873	−2.515	−2.448	−2.171
*p*-value	0.005^b^	0.009^b^	**0.011**	0.027^b^
FIGO stage				
IA	3.48 (3.22–3.66)	1.00 ± 0.10	0.77 (0.75–0.81)	1.22 ± 0.10
IIB	3.78 (3.52–4.14)	0.95 ± 0.06	0.84 (0.81–0.87)	1.12 ± 0.11
*t*/*z* value	−3.535	2.873	−5.181	3.524
*p*-value	<0.001^a^	0.005^b^	<0.001^a^	0.001^b^
Lymphovascular space invasion				
Positive	3.85 ± 0.34	0.94 ± 0.06	0.83 (0.80–0.86)	1.13 ± 0.11
Negative	3.48 ± 0.40	0.99 ± 0.09	0.78 (0.75–0.82)	1.20 ± 0.10
*t*/*z* value	3.889	−2.521	−3.375	2.604
*p*-value	<0.001^b^	0.014^b^	0.001^a^	0.015^b^
Histological grade				
Non-high grade (grades I and II)	3.46 ± 0.38	0.98 (0.92–1.04)	0.78 (0.75–0.81)	1.20 ± 0.10
High grade (grade III)	3.92 ± 0.32	0.93 (0.90–0.99)	0.86 (0.83–0.87)	1.12 ± 0.11
*t*/*z* value	−5.030	−2.361	−4.999	2.582
*p*-value	<0.001^b^	0.018^a^	<0.001^a^	0.016^b^
Risk stratification				
Low risk	3.41 (3.14–3.64)	1.01 ± 0.09	0.76 (0.74–0.80)	1.23 ± 0.09
Non-low risk (intermediate, high-intermediate, and high)	3.77 (3.51–4.13)	0.94 ± 0.06	0.83 (0.80–0.86)	1.12 ± 0.11
*t*/*z* value	−4.055	3.261	−5.101	4.328
*p*-value	<0.001^a^	0.002^b^	<0.001^a^	<0.001^b^

Values shown in bold denote statistical significance in the comparison.

MTRasym, magnetization transfer ratio asymmetry; ADC, apparent diffusion coefficient; MK, mean kurtosis; MD, mean diffusivity.

a Comparisons performed using Mann–Whitney U test.

b Comparisons performed using independent t-test.

**Table 4:** Predictive performance of the different parameters.

Parameters	AUC (95% CI)	*p*-value	Cutoff	Sensitivity (%)	Specificity (%)	Youden index (%)
Histological subtype						
MTRasym (3.5 ppm) (%)	0.797 (0.686–0.883)	<0.001	3.780	77.61	80.00	57.61
ADC (×10^−3^ mm^2^/s)	0.839 (0.733–0.915)	<0.001	0.928	71.64	100.00	71.64
MK	0.830 (0.723–0.908)	<0.001	0.831	76.12	80.00	56.12
MD (×10^−3^ mm^2^/s)	0.793 (0.681–0.879)	<0.001	1.159	64.18	100.00	64.18
FIGO stage						
MTRasym (3.5 ppm) (%)	0.748 (0.632–0.843)	<0.001	3.750	86.36	53.57	39.94
ADC (×10^−3^ mm^2^/s)	0.665 (0.544–0.772)	0.011	1.043	29.55	96.43	25.97
MK	0.864 (0.763–0.933)	<0.001	0.784	68.18	96.43	64.61
MD (×10^−3^ mm^2^/s)	0.723 (0.605–0.822)	<0.001	1.233	50.00	85.71	35.71
Lymphovascular space invasion						
MTRasym (3.5 ppm) (%)	0.775 (0.662–0.865)	<0.001	3.520	57.41	94.44	51.85
ADC (×10^−3^ mm^2^/s)	0.693 (0.574–0.797)	0.003	0.993	48.15	88.89	37.04
MK	0.767 (0.652–0.859)	<0.001	0.784	55.56	94.44	50.00
MD (×10^−3^ mm^2^/s)	0.698 (0.578–0.801)	0.006	1.213	55.56	77.78	33.33
Histologic grade						
MTRasym (3.5ppm) (%)	0.828 (0.721–0.907)	<0.001	3.750	83.64	70.59	54.22
ADC (×10^−3^ mm^2^/s)	0.690 (0.570–0.794)	0.007	1.036	29.09	94.12	23.21
MK	0.903 (0.810–0.960)	<0.001	0.815	78.18	94.12	72.30
MD (×10^−3^ mm^2^/s)	0.693 (0.573–0.796)	0.006	1.217	50.91	76.47	27.38
Risk stratification						
MTRasym (3.5 ppm) (%)	0.780 (0.667–0.870)	<0.001	3.490	83.87	56.10	39.97
ADC (×10^−3^ mm^2^/s)	0.709 (0.590–0.810)	<0.001	0.915	41.94	87.80	29.74
MK	0.853 (0.750–0.925)	<0.001	0.784	93.55	70.73	64.28
MD (×10^−3^ mm^2^/s)	0.766 (0.652–0.858)	<0.001	1.233	87.10	53.66	40.76
Combined diagnosis	0.906 (0.814–0.962)	<0.001	–	70.97	92.68	63.65

The combined diagnosis represents MTRasym (3.5 ppm) + D + MK.

AUC, area under the receiver operating characteristic curve; MTRasym, magnetization transfer ratio asymmetry; ADC, apparent diffusion coefficient; MK, mean kurtosis; MD, mean diffusivity.

The results for the differentiation of stages IA and IB in stage I EC are shown in **Tables 3**, **4** and **Figure 3B**. Significantly greater MTRasym (3.5 ppm) and MK but significantly lower ADC and MD were found in the stage IA group compared with those in the stage IB group (all *p* < 0.05). The AUCs of ADC, MD, MTRasym (3.5 ppm), and MK were 0.665, 0.723, 0.748, and 0.864, respectively, and there were significant differences between the AUCs of MK and ADC and between the AUCs MK and MD (*Z* = 2.779 and 2.074, *p* = 0.006 and 0.04, respectively).

Compared with the quantification of the LVSI-negative group in stage I EC, the MTRasym (3.5 ppm) and MK in the LVSI-positive groups were significantly greater and the ADC and MD were significantly lower (all *p* < 0.05). The AUCs of ADC, MD, MK, and MTRasym (3.5 ppm) were 0.693, 0.698, 0.767, and 0.775, respectively, with no significant difference for each between the LVSI-negative and LVSI-positive groups (**Tables 3**, **4** and **Figure 3C**).

Quantification of the high-grade group in stage I EC was for grade III, while that of the non-high-grade group was for grades I and II. MTRasym (3.5 ppm) and MK were found significantly greater while ADC and MD were significantly lower in the high-grade group than those in the non-high-grade group (all *p* < 0.05). The AUCs of ADC, MD, MTRasym (3.5 ppm), and MK were 0.690, 0.693, 0.828, and 0.903, respectively, and significant differences were found between the AUCs of MK and ADC and between the AUCs of MK and MD (*Z* = 2.625 and 2.974, *p* = 0.008 and 0.003, respectively) (**Tables 3**, **4** and **Figure 3D**).

### Assessment of Risk Stratification

**Figure 4:**
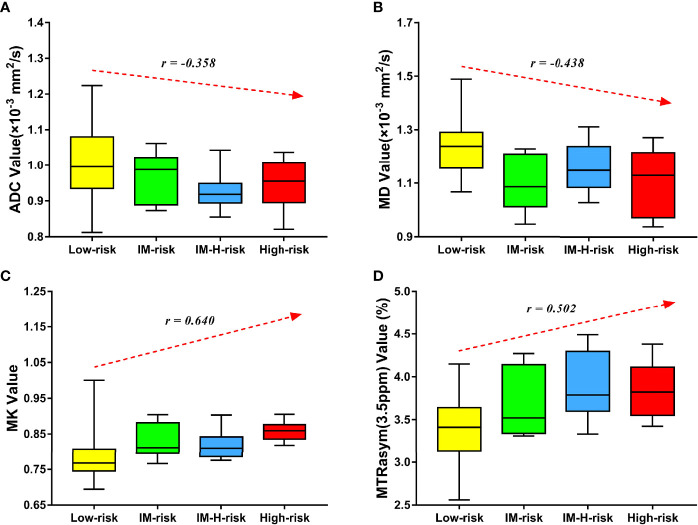
Correlation of various parameters with risk stratification (*LM*, intermediate; *LM-H*, intermediate-high). The apparent diffusion coefficient (ADC) **(A)** and mean diffusivity (MD) **(B)** were mildly negatively correlated with risk stratification (*r* = −0.358 and −0.438, respectively). The mean kurtosis (MK) **(C)** and magnetization transfer ratio asymmetry (MTRasym, at 3.5 ppm) **(D)** were moderately positively correlated with risk stratification (*r* = 0.640 and 0.502, respectively).

MK and MTRasym (3.5 ppm) were moderately positively correlated with risk stratification, with *r* values of 0.640 (95% CI = 0.479–0.759, *p* < 0.001) and 0.502 (95% CI = 0.306–0.657, *p* < 0.001), respectively. ADC and MD were mildly negatively correlated with risk stratification, with *r* values of −0.358 (95% CI = −0.544 to −0.138, *p* = 0.002) and −0.438 (95% CI = −0.608 to −0.229, *p* < 0.001), respectively (**Figure 4**). Only the difference in the *r* values between MK and ADC was statistically significant (*Z* = 2.253, *p* = 0.024) (**Figure 4**).

**Figure 5:**
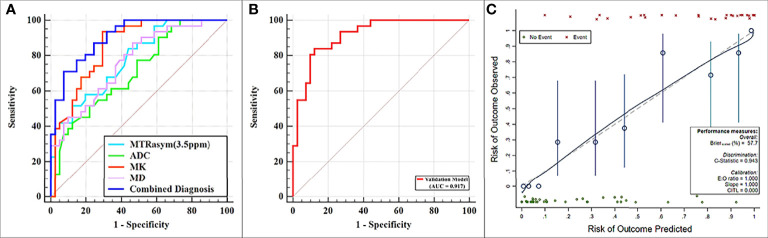
**(A)** Receiver operating characteristic (ROC) curves showing each parameter and the combination of mean diffusivity (MD), mean kurtosis (MK), and magnetization transfer ratio asymmetry (MTRasym, at 3.5 ppm) for the differentiation of low- and non-low-risk stage I endometrial cancer (EC) patients. **(B, C)** ROC and calibration curves for predicting risk stratification of stage I EC patients in the validation model.

MTRasym (3.5 ppm) and MK were significantly greater while ADC and MD were significantly lower in the non-low-risk group than those in the low-risk group (all *p* < 0.05) (**Table 3**). The AUCs of ADC, MD, MTRasym (3.5 ppm), and MK increased successively, which were 0.709, 0.766, 0.780, and 0.853, respectively, but only the differences between the AUCs of MK and ADC were significant (*Z* = 1.981, *p* = 0.047) (**Tables 3**, **4** and **Figure 5A**).

The potential risk-related factors of age, tumor size, ADC, MD, MK, and MTRasym (3.5 ppm) were investigated in the logistic regression analysis to explore their value for the stratification of low- and non-low-risk stage I EC patients.

Univariate analysis showed statistical significance for ADC, MTRasym (3.5 ppm), MD, and MK as risk predictors (*p*-values of 0.004, <0.001, <0.001, and <0.001, respectively), while multivariate analysis revealed that MTRasym (3.5 ppm), MK, and MD were independent predictors (*p*-values of 0.005, 0.034, and 0.015, respectively).

The combination of the independent predictors [MD, MK, and MTRasym (3.5 ppm)] showed optimal predictive performance (AUC = 0.906, sensitivity = 70.97%, specificity = 92.68%, *p* < 0.001), which was significantly better than those of ADC (AUC = 0.709, *Z* = 3.013, *p* = 0.003), MTRasym (3.5 ppm) (AUC = 0.780, *Z* = 2.852, *p* = 0.004), and MD (AUC = 0.766, *Z* = 2.787, *p* = 0.005) individually, but not MK (AUC = 0.853, *Z* = 1.414, *p* = 0.157) (**Table 5** and **Figure 5A**).

**Table 5:** Univariate and multivariate analyses for the identification of low- and non-low-risk EC patients.

Parameters	Univariate analyses		Multivariate analyses	
	OR (95% CI)	*p*-value	OR (95% CI)	*p*-value
Age (years)	1.386^a^ (0.855–2.247)	0.186	–	–
Tumor size (mm)	1.445^a^ (0.885–2.393)	0.139	–	–
MTRasym (3.5 ppm) (%)	4.334^a^ (2.009–9.349)	<0.001	3.897^a^ (1.501–10.119)	**0.005**
ADC (×10^−3^ mm^2^/s)	0.402^a^ (0.215–0.750)	0.004	0.524^a^ (0.220–1.249)	0.145
MK	4.528^a^ (2.113–9.704)	<0.001	2.781^a^ (1.083–7.142)	**0.034**
MD	0.304^a^ (0.158–0.585)	<0.001	0.312^a^ (0.121–0.799)	**0.015**

Values in bold are statistically significant.

OR, odds ratio; CI, confidence interval; MTRasym, magnetization transfer ratio asymmetry; ADC, apparent diffusion coefficient; MK, mean kurtosis; MD, mean diffusivity.

a OR per 1 standard deviation.

The calibration curves generated by the analysis of bootstrapped samples are shown in **Figures 5B, C** and were used to validate the multi-parameter regression model that included MD, MK, and MTRasym (3.5 ppm). There was high consistency between the predicted and the observed risk stratification for stage I EC.

## Discussion

Both ADC and MD can be used to reflect the degree of the restricted diffusion movement of water molecules in tissues. Generally, the higher the density of tissue cells, the more significant the limitation of the diffusion movement of water molecules and, thus, the smaller the ADC and MD (11, 12). ADC and MD have been used to evaluate stage I EC in several studies. The study of An et al. showed that the ADC histogram was conducive to the evaluation of stage I EC histological subtype, grade, FIGO stage, and even risk stratification (18). Meng et al. used the average ADC and MD based on the total tumor volume for stage I EC risk stratification assessment, and the results showed that these values decreased with the increase in risk stratification; significant differences in the ADC and MD between the low-risk and non-low-risk groups were observed (19). In the present study, the ADC and MD were lower in the non-adenocarcinoma, stage IB, high-grade, and non-low-risk groups than those in the adenocarcinoma, stage IA, non-high-grade, and low-risk groups (all *p* < 0.05), which was generally consistent with the above findings. The results of both ADC and MD being lower in the LVSI-positive group than those in the LVSI-negative group were similar to the findings of Ma et al. (24) and Yamada et al. (13), suggesting that both parameters can be helpful for LVSI assessment in stage I EC. Due to the tighter tissue structure in patients of the LVSI-positive group, a more significant restriction of water molecule diffusion within it may be the main reason for the above results. MD rather than ADC in the multivariate regression analysis was found as an independent predictor, which might be related to the fact that MD was calculated by taking into account the restricted diffusion of water molecules in all directions and therefore could assess the diffusion of water molecules more accurately than ADC (12, 15).

MK is a representative parameter of DKI that is mainly used to reflect the degree of deviation from the Gaussian distribution of water molecule diffusion movement in tissues (12). Usually, malignant lesions with complex tissue structures are assumed to have a higher degree of deviation from the Gaussian distribution of water molecule diffusion movement, which means larger MK values (13, 19). Previous studies have shown that MK can provide a valid assessment of the histological type, grade, stage, and LVSI status of EC patients due to differences in the cell density, nuclear heterogeneity, and other factors (13, 24–26). However, these studies included patients with different FIGO stages of EC, so it may be difficult to provide a more definitive reference for the management of patients with stage I EC. Our results for MK in stage I EC patients for the groups in histological subtypes, grades, FIGO stages, and even LVSI status were similar to those described above. The results also showed that MK was not only effective in assessing the above risk factors but also one of the independent predictors for discriminating between non-low-risk and low-risk stage I EC patients, which was consistent with the results of the study conducted by Meng et al. (19) using the old ESMO clinical practice guidelines, indicating that MK can play a reliable role in the risk assessment of stage I EC patients.

APTWI is a MRI molecular imaging technique, and MTRasym (3.5 ppm) characterizes the heterogeneous metabolism of mobile proteins and peptides due to changes in the histopathology and genetic expression of tumors (14, 27, 28). Previous investigations have revealed that a higher MTRasym (3.5 ppm) indicated a high level of mobile protein and peptide metabolism, which was associated with more active cell proliferation, more microscopic necrosis (29), greater microvascular density (30), and an appropriate pH level (31). Only a few studies have explored the value of APTWI for the assessment of EC. The study by Takayama et al. showed that MTRasym (3.5 ppm) was positively correlated with the histological grade of endometrial adenocarcinoma (16), and the work by Meng et al. revealed that MTRasym (3.5 ppm) can be used to differentiate EC of different clinical types, histological grades, subtypes, and risk stratification (17, 19). In the present study, MTRasym (3.5 ppm) showed similar performance to that in the aforementioned studies in the identification of stage I EC patients with different histological subtypes, grades, and risk stratification. To our knowledge, our study is the first to conduct the evaluation of APTWI for identifying stage I EC patients with different FIGO stages and LVSI status. The higher MTRasym (3.5 ppm) in the stage IB and LVSI-positive groups was speculated to be related to the fact that the EC in these groups has more active cell proliferation, which leads to an increased content of mobile protein peptides in the tissues (13, 25).

Several limitations of this research should be taken into account. Firstly, our study was designed at a single institution with a relatively small number of patients, which may have contributed to selection bias. Secondly, both APTWI and DKI based on echo planar imaging acquisition had poor signal-to-noise ratios and low spatial resolution, making the assessment of small EC lesions difficult (largest area, <50 pixels). Thirdly, the APTWI sequence used in the present study was two-dimensional, and although we replicated the position, layer spacing, and layer thickness of the previous sequence layer by layer throughout the scanning procedure, this not only led to an increase in the scanning time but also may have introduced errors. In the future, we will include a larger population, attempt to conduct multi-institutional studies, and refine the relevant scanning techniques to make the findings more complete and reliable.

## Conclusion

Although a similar performance was obtained with each single parameter of APTWI, DWI, and DKI for the noninvasive assessment of aggressive behavior in stage I EC, the combination of MD, MK, and MTRasym (3.5 ppm) provided improved predictive power for non-low-risk stage I EC and may serve as a superior imaging marker.

## Data Availability Statement

The raw data supporting the conclusions of this paper will be made available by the authors, without undue reservation.

## Ethics Statement

The studies involving human participants were reviewed and approved by the First Affiliated Hospital of Xinxiang Medical University. The patients/participants provided written informed consent to participate in this study.

## Author Contributions

DH: study concepts, and design. XJ, ZL, and GZ: literature research. WL and HW: clinical studies. MZ and RY: data analysis. XJ and ZL: manuscript preparation. JG and KW: manuscript editing. All authors contributed to the article and approved the submitted version.

## Funding

This work was supported by the Roentgen Imaging Research Project (HN-20201017-002) and the Key Project of Henan Province Medical Science and Technology Project (LHGJ20200519).

## Conflict of Interest

JG and KW were employed by GE Healthcare.

The remaining authors declare that the research was conducted in the absence of any commercial or financial relationships that could be construed as a potential conflict of interest.

## Publisher’s Note

All claims expressed in this article are solely those of the authors and do not necessarily represent those of their affiliated organizations, or those of the publisher, the editors and the reviewers. Any product that may be evaluated in this article, or claim that may be made by its manufacturer, is not guaranteed or endorsed by the publisher.
